# Genetic diversity, population structure, and phylogeny of insular Spanish pepper landraces (*Capsicum annuum* L.) through phenotyping and genotyping-by-sequencing

**DOI:** 10.3389/fpls.2024.1435427

**Published:** 2024-10-30

**Authors:** Neus Ortega-Albero, Lorenzo Barchi, Ana Fita, Miguel Díaz, Felipe Martínez, Joana-Maria Luna-Prohens, Adrián Rodríguez-Burruezo

**Affiliations:** ^1^ Institute for the Conservation and Improvement of Valencian Agrodiversity (COMAV), Universitat Politècnica de València, València, Spain; ^2^ Department of Agricultural, Forest and Food Sciences (DISAFA), Plant Genetics, University of Torino, Grugliasco, Italy; ^3^ Department of Vegetal Production, Institut de Recerca i Formació Agroalimentària i Pesquera de les Illes Balears (IRFAP), Palma, Spain

**Keywords:** agrodiversity, cultivated endemism, genetic background, heterozygosity, phenomics, single nucleotide polymorphism, vegetable crops

## Abstract

Pepper (*Capsicum* spp.) is one of the most important crops worldwide. Understanding the species’ genetic background is key to preserve agrodiversity on-farm, to contribute to a more diverse and resilient agrifood sector, and to find new sources of variation that could be useful in future breeding programs. In this regard, varietal groups bred in insular environments have gained special interest as they have evolved quite isolated from continental forms, with a limited genetic exchange. The present work explores the diversity of a plethora of Balearic landraces, corresponding to different local varietal types, through phenotyping and genotyping-by-sequencing (GBS). Mallorca and Eivissa landraces were phenotyped according to a comprehensive list of descriptors for plant, leaf, flower, fruit, pollen, and seed and were genotyped with single nucleotide polymorphism (SNP) markers; population structure and their patterns of diversity were studied. The results showed a considerable morphological diversity for most traits analyzed, within and between landraces. On the whole, in regard to genetic patterns, relatively low levels of heterozygosity and moderate genetic diversity for the studied landraces were found although some of them exhibited diverse patterns. The materials were not grouped in specific clusters associated with each island, but mainly according to varietal types. These findings can serve as the basis for studying divergent evolutionary patterns associated with the corresponding populations. Finally, the results can contribute to further elucidation of the genetic basis of Balearic landraces and serve as an inspiring case of study for other insular endemisms of cultivated species.

## 1 Introduction

Peppers and chilies, among other terms, are recognizable names for *Capsicum* spp., one of the most important cultivated vegetables in the world for fresh consumption and as a spice with more than 40 Mt of fruit production and 3 Mha of harvested area (Jarret et al., 2019; FAO, 2023). More than 30 species are compressed in the *Capsicum* genus, from which only five are recognized as cultivated species, namely, *Capsicum annuum* L., *C. baccatum* L., *C. chinense* Jacq., *C. frutescens* L., and *C. pubescens* Ruiz & Pav. Among them, *C. annuum* is the most diverse and economically relevant including well-known varietal types like ‘bell,’ ‘jalapenos,’ ‘numex,’ and ‘ancho’ (Pickersgill, 1991; Bartolomé Garcia et al., 2015; Pereira-Dias et al., 2019, 2020).

*Capsicum* cultivated species present a broad diversity in morphological and agronomic traits mostly in Central and South America, their geographical origin, as a result of evolution, domestication, and artificial and natural selection in primary and secondary centers of diversity (DeWitt and Bosland, 1996; Bartolomé Garcia et al., 2015; Tripodi et al., 2021). Indeed, more than five centuries of pepper selection in Europe have resulted in a great range of landraces adapted to a wide range of agroclimatic conditions (Bartolomé Garcia et al., 2015).

Spain is considered an important secondary center of diversity of peppers that were brought into the country from Mexico since the XV century as an alternative to the Asian black pepper (*Piper nigrum* L.). In this regard, Spain has the largest number of European Union Protected Designations of Origin (PDO) such as, among others, ‘Pemento de Herbón’ and ‘Pimiento del Piquillo de Lodosa’ and Protected Geographical Indications (PGIs) such as ‘Pemento do Couto’ and ‘Pimiento asado del Bierzo,’ and many local varieties can be found on each Spanish region, including the Canary Islands and the Balearic Islands (Rodríguez-Burruezo et al., 2016).

In this context, the demands of the consumers for improved tastes, or the recovery of the “old ones,” and the need to develop resilient vegetables adapted to diseases and changing environments are the main reasons why landraces are being reintroduced into the market. Moreover, these varieties have great importance because they are available, preserved *in-situ* materials for conservation and breeding programs that may help to revert agrodiversity loss due to genetic erosion. They have also proved to be key for farmers and communities’ rights preservation and empowerment (Lanteri et al., 2003; Parisi et al., 2007; Brugarolas and Ruiz, 2009; Rodríguez-Burruezo et al., 2016). In this frame, farmers from the Balearic Islands Mallorca (Majorca) and Eivissa (Ibiza) have been very active in growing and preserving endemic landraces for centuries. As “living laboratories,” these high added value landraces are still grown and preserved by these farmers in their socioeconomic environment, constantly adapting and evolving to insular environments, helping to the study of evolution processes (Gillespie and Baldwin, 2010). Moreover, landraces have proved to be useful for understanding and predicting how species may respond to uncertain climate changes (Emerson, 2002; Farrell et al., 2015).

The knowledge on the available germplasm diversity, phylogenetic relations between accessions, and the genetic basis of the adaptation to specific conditions are major challenges for establishing pepper breeding programs (Prohens et al., 2017). However, despite its importance, genetic information for *Capsicum* has been obtained later than other *Solanaceae* species such as tomato and potato, mainly due to its large genome (3.5 Gb) and the high amount of repeated elements (70%–80%) (Ashrafi et al., 2012; Qin et al., 2014). Through the last decades, the development of high-throughput sequencing technologies allowed us to obtain fast and low-cost data (Metzker, 2010; Edwards et al., 2013). Among them, genotyping-by-sequencing (GBS) plays an important role in new scientific insights across species or populations, particularly for its relative simplicity (Elshire et al., 2011; Poland and Rife, 2012). Thus, GBS has been recently used in *Capsicum* peppers for studies focused on germplasm diversity, population structure, and genomic analysis as a result of domestication or local adaptation (Taranto et al., 2016; Pereira-Dias et al., 2019; Chae et al., 2022).

Here, we present a genomic characterization performed with GBS and a morphological characterization, using conventional descriptors of a collection of pepper landraces from Mallorca and Eivissa. Our goals were to analyze i) the morphological variation of insular landraces to contribute to their varietal typification, promotion, and preservation; ii) the genetic relations of a collection of Balearic landraces, historically conserved and multiplied *in situ* by farmers; and iii) the genetic patterns of Balearic landraces, specifically adapted to insular conditions.

## 2 Materials and methods

### 2.1 Plant material, DNA extraction, library preparation, and sequencing

**Figure 1:**
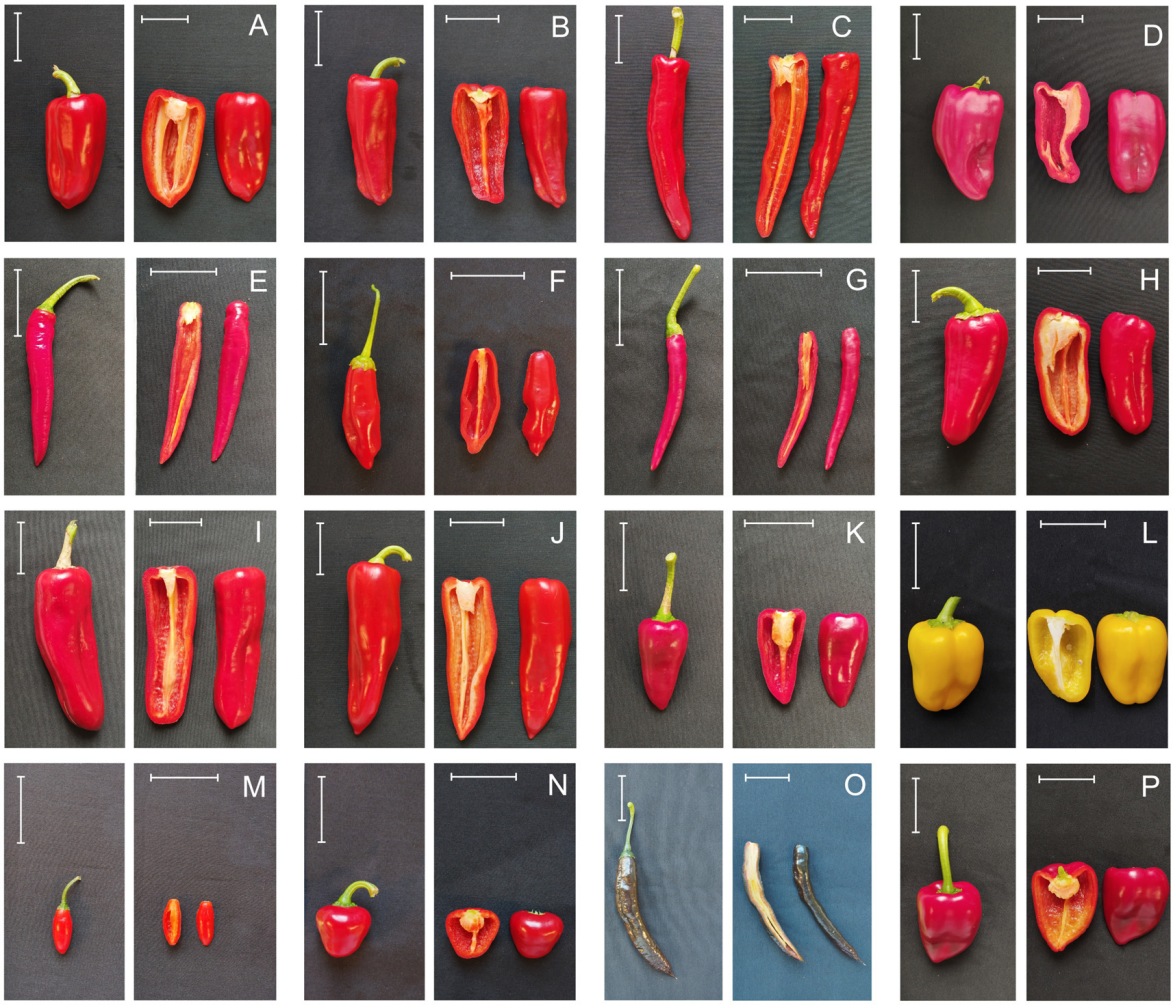
Illustration of the varietal types evaluated. Pebrera Blanca **(A)**, Citró de Matances **(B)**, Banya de Cabra **(C)**, Blau **(D)**, Cirereta **(E)**, D’Envinagrar **(F)**, Fulla d’Olivera **(G)**, Ros **(H)**, Ros Gruixat **(I)**, Ros Prim **(J)**, Tap de Cortí **(K)**, California Wonder **(L)**, Chile Serrano **(M)**, Bola **(N)**, Pasilla Bajío **(O)**, and Piquillo **(P)**. The white bar indicates 5 cm length.

Seeds from 73 accessions corresponding to 11 landraces from Mallorca and Eivissa, plus six reference varieties used as external controls and representing a wide variability of *Capsicum* morphologies, were sown in seedling trays in January 2023 (**Figure 1**; **Table 1**; **Supplementary Table S1**). Young tissue samples were taken from one plantlet per accession at the four-leaf stage, before transplanting. DNA was then extracted from 100 mg of deep-frozen leaf tissue with the SILEX method, developed by Vilanova et al. (2020).

**Table 1:** Studied varietal types, with the corresponding abbreviations, origins, and number of phenotyped and genotyped accessions.

Varietal type/local name	Abbreviation	Number of phenotyped accessions	Number of genotyped accessions
*Eivissa/Ibiza*			
Pebrera Blanca	PB	6	26
Citró de Matances	CdM	9	21
*Mallorca/Majorca*			
Banya de Cabra	BdC	3	2
Blau	B	3	2
Cirereta	C	1	1
D’Envinagrar	DE	2	1
Fulla d’Olivera	FO	3	2
Ros	R	3	2
Ros Gruixat	RG	4	3
Ros Prim	RP	2	2
Tap de Cortí	TdC	5	5
*Reference controls*			
California Wonder (breeding line, Spain)	CW	1	1
Chile Serrano (Mexico)	CS	1	1
Bola (Murcia, Spain)	BOL	1	1
Pasilla Bajío (Mexico)	PAS	1	1
Serrano Criollo de Morelos (Mexico)	SCM	1	1
Piquillo (Navarra, Spain)	PIQ	1	1

DNA digestion was performed from 100 ng of genomic DNA with ApeKI enzyme and 3.6 mg of adapters. GBS library construction and sequencing were carried out by The Elshire Group Ltd. (Palmerston North, New Zealand) following the protocol by Elshire et al. (2011), including library amplification with 18 PCR cycles and sequencing with Illumina NovaSeq 6000 platform, using paired-end 150 pb reads.

### 2.2 Mapping and SNP calling

Raw reads were demultiplexed and barcodes were removed using Kevin-Murray’s AX-demux algorithm (Murray and Borevitz, 2018). Demultiplexed reads were aligned to the reference *C. annuum* genome CM334 (Criollo de Morelos version 1.55) (Kim et al., 2014) using BWA-mem (Li, 2013) with default parameters except for avoiding multiple-mapping reads. GATK haplotype-caller 4.1.9 (Depristo et al., 2011) was used to perform the SNP calling on the BAM files and select SNPs with minimum mapping quality (*Q* > 30). VCFtools (Danecek et al., 2011) with missing data <15% and mean minimal read depth >7 was used for selecting good quality SNPs. Finally, heterozygosity was also calculated for each accession with VCFtools (Danecek et al., 2011). For further analysis, SNPs with missing data per site <25% and MAF >5% were retained with bcftools (Li, 2011) and plink2 (Chang et al., 2015).

### 2.3 Evaluation of phenotypic traits and statistical treatment

To assess the phenotypic variation in the Balearic collection, 41 accessions representing the group of target landraces and the six control varieties were transplanted in March 2023 to 30 cm diameter pots with coconut fiber, under experimental greenhouse conditions of the Universitat Politècnica de València at the Campus de Vera (València, Spain) (**Table 1**). Plants were trained with vertical strings, drip-irrigated, and fertilized by fertigation, following the standard practices for this crop.

Phenotyping was performed in one plant per accession in the Spring–Summer season 2023, using 29 descriptors for *Capsicum*, related to plant (6), inflorescence/flower (6), fruit (14), pollen (2), and seeds (1) as described on **Table 2**, including their abbreviations to facilitate their presentation in the rest of tables and figures. Most traits corresponded to *Capsicum* descriptors from Bioversity International (International Plant Genetic Resources Institute, IPGRI, 1995), and fruit wall consistency and pollen morphological and biological viability were included as commonly used traits for germplasm characterization in *Capsicum*.

**Table 2:** List of qualitative and quantitative traits measured, corresponding abbreviation, IPGRI descriptor number, and units or scale, as necessary.

Descriptor	Abbreviation	IPGRI number	Units/scale
*Plant descriptors*			
Stem color	SC	7.1.2.2	1 = green, 2 = green with purple stripes, 3 = purple
Nodal anthocyanin	NA	7.1.2.3	1 = green, 3 = light purple, 5 = purple, 7 = dark purple
Stem pubescence	SP	7.1.2.5	3 = sparse, 5 = intermediate, 7 = dense
Stem length	SL	7.1.2.9	mm
Leaf color	LC	7.1.2.14	1 = yellow, 2 = light green, 3 = green, 4 = dark green, 5 = light purple, 6 = purple, 7 = variegated
Leaf shape	LS	7.1.2.15	1 = deltoid, 2 = ovate, 3 = lanceolate
*Inflorescence/flower descriptors*			
Number of flowers per axil	NFA	7.2.1.2	–
Flower position	FP	7.2.1.3	3 = pendant, 5 = intermediate, 7 = erect
Anther color	AC	7.2.1.8	1 = white, 2 = yellow, 3 = pale blue, 4 = blue, 5 = purple
Filament color	FC	7.2.1.10	1 = white, 2 = yellow, 3 = green, 4 = blue, 5 = light purple, 6 = purple
Stigma exsertion	SE	7.2.1.12	3 = inserted, 5 = same level, 7 = exserted
Calyx margin	CM	7.2.1.15	1 = entire, 3 = intermediate, 5 = dentate
*Fruit descriptors*			
Anthocyanin spots or stripes	AS	7.2.2.2	0 = absent, 1 = present
Fruit color at intermediate stage	FCI	7.2.2.3	3 = light green, 5 = green, 7 = dark green
Fruit color at mature stage	FCM	7.2.2.6	1 = white, 2 = lemon-yellow, 3 = pale orange-yellow, 4 = orange-yellow, 5 = pale orange, 6 = orange, 7 = light red, 8 = red, 9 = dark red, 10 = purple, 11 = brown, 12 = black
Fruit color lightness (L)	FL	–	0 = black to 100 = white
Fruit color green/red value (a)	FA	–	Negative = green to positive = red
Fruit color blue/yellow value (b)	FB	–	Negative = blue to positive = yellow
Fruit shape	FS	7.2.2.7	1 = elongate, 2 = almost round, 3 = triangular, 4 = campanulate, 5 = blocky
Fruit length	FLE	7.2.2.8	mm
Fruit width	FWI	7.2.2.9	mm
Fruit weight	FW	7.2.2.10	g
Fruit shape at blossom end	FSBE	7.2.2.15	1 = pointed, 2 = blunt, 3 = sunken, 4 = sunken and pointed
Number of locules	NL	7.2.2.18	–
Pedicel with ripe fruit persistence	PP	7.2.2.20.2	3 = slight, 5 = intermediate, 7 = persistent
Fruit wall consistency	FWC	–	0 = non-consistent, 1 = consistent
*Pollen descriptors*			
Pollen morphological viability	PMV	–	–
Pollen biological viability	PBV	–	–
*Seed descriptors*			
100-seed weight	SW	7.3.5	g

Fruit wall consistency was estimated by applying pressure to the pericarp with the hand. We considered consistent fruit walls when the fruit did not deform permanently after finger pressure, while non-consistent or soft fruit walls were those which lost their shape permanently after pressure and/or showed permanent bruising. Pollen morphological and biological viabilities were measured using a light microscope SE (Nikon Instruments Inc., Melville, NY, USA), with pollen samples from four flowers per plant, dyed with tetrazolium (Norton, 1966; Rodriguez-riano and Dafni, 2000; Manzur et al., 2015). Pollen samples were kept for 15 min in the darkness, after which four fields were observed per sample, with a total magnification of ×100. Morphological viability was estimated as the ratio between turgent grains against total grains (turgent + deformed grains). Biological viability was obtained in the same way, but only considering turgent tetrazolium-dyed grains against total grains (turgent-dyed + turgent-non-dyed + deformed). Each pollen viability value corresponds to the average of four fields in each sample.

One plant per accession, representative of the accession and whose DNA was previously extracted, was characterized. For qualitative traits, one observation was made to establish a unique genotype–phenotype relationship. For fruit quantitative traits, five measurements per plant were made. Fruit color was measured using a Minolta CR-300 colorimeter (Minolta Corporation, Osaka, Japan) and expressed according to CIE L*a*b* 1976 space. For this fruit trait, 10 measures per accession were taken in homogeneous spots, i.e., two measures per fruit in five fruits. In a few accessions, no fruits with enough quality were obtained due to their lack of adaptation to the experimental conditions of this work. Therefore, in such cases, it was not possible to present the results of the corresponding traits.

Phenotypic data were normalized, and outliers were removed. For qualitative traits, the percentages of each trait level were calculated per accession and per landrace. Statgraphics Centurion XVII software (StatPoint Technologies, Warrenton, VA, USA) was used to estimate means and standard deviations for each quantitative trait, as well as to perform principal component analysis (PCA) with both qualitative and quantitative traits. Finally, intertrait correlations were calculated with all phenotypic data and Pearson coefficient <0.05.

### 2.4 SNP diversity analysis

To describe the structure of the population, a PCA was performed with Rpackage SNPrelate (Zheng et al., 2012). To elucidate the relationship between individuals, a phylogenetic tree was obtained with IQ-TREE (Minh et al., 2020), and a kinship matrix was obtained with SNPrelate (Zheng et al., 2012). An admixture analysis was built with AdminPipe v2.0 (Mussmann et al., 2020) to understand the genomic admixture of the studied population. For the analysis, 10 subpopulations (*K*) were used and each *K* run was statistically analyzed with Pong statistical software (Behr et al., 2016) using 19 replicates to obtain the best statistical run.

## 3 Results and discussion

### 3.1 Study of phenotypic variation

#### 3.1.1 Qualitative traits

On the whole, a considerable diversity was found in both the reference group of control varieties and the collection of Balearic accessions for most traits. By contrast, some traits were found monomorphic, in both the reference group and Balearic accessions, like stem color and fruit wall consistency (**Tables 3**, **4**).

**Table 3:** Phenotypic values in plant and flower qualitative traits of the landraces and the reference varieties.

Varietal type/local name	SC	NA	SP	NFA	FP	AC	FC	SE	CM
Pebrera Blanca	Green	Light purple (17%) Purple (83%)	Sparse	1	Intermediate (60%) Erect (40%)	Blue (50%) Purple (50%)	White	Inserted (50%) Same level (33%) Exserted (17%)	Intermediate
Citró de Matances	Green (89%) Purple (11%)	Light purple (22%) Purple (78%)	Sparse (78%) Intermediate (11%) Dense (11%)	1 (89%) 2 (11%)	Intermediate (33%) Erect (67%)	Blue (67%) Purple (33%)	White	Inserted (11%) Same level (33%) Exserted (56%)	Enture (11%) Intermediate (78%) Dentate (11%)
Banya de Cabra	Green	Purple	Sparse	1 (67%) 2 (33%)	Pendant (33%) Intermediate (33%) Erect (33%)	Blue	White (67%) Light purple (33%)	Same level (33%) Exserted (67%)	Intermediate (67%) Dentate (33%)
Blau	Green	Purple	Sparse	1 (67%) 2 (33%)	Intermediate (67%) Erect (33%)	Blue (33%) Purple (67%)	White (67%) Light purple (33%)	Inserted (33%) Same level (33%) Exserted (33%)	Intermediate
Cirereta	Green	Purple	Sparse	1	Erect	Purple	White	Same level	Intermediate
D’Envinagrar	Green	Light purple	Sparse	1	Intermediate	Yellow (50%) Purple (50%)	White	Inserted	Dentate
Fulla d’Olivera	Green	Light purple (33%) Purple (67%)	Sparse	1	Erect	Blue	White	Same level (33%) Exserted (67%)	Intermediate (67%) Dentate (33%)
Ros	Green	Light purple (33%) Purple (67%)	Sparse	1 (67%) 2 (33%)	Intermediate (67%) Erect (33%)	Blue	White	Same level (67%) Exserted (33%)	Intermediate
Ros gruixat	Green	Purple	Sparse	1	Intermediate (75%) Erect (25%)	Blue (75%) Purple (25%)	White	Inserted (50%) Same level (50%)	Intermediate (75%) Dentate (25%)
Ros prim	Green	Purple	Sparse	1	Intermediate	Blue (50%) Purple (50%)	White	Inserted (50%) Same level (50%)	Intermediate (50%) Dentate (50%)
Tap de cortí	Green	Green (20%) Purple (80%)	Sparse	1 (40%) 2 (60%)	Erect	Pale blue (20%) Blue (40%) Purple (40%)	White	Inserted (20%) Same level (60%) Exserted (20%)	Intermediate
California Wonder	Green	Light purple	Sparse	1	Pendant	Blue	White	Inserted	Intermediate
Chile Serrano	Green	Light purple	Intermediate	1	Pendant	Purple	White	Exserted	Dentate
Bola	Green	Purple	Sparse	1	Pendant	Blue	White	Inserted	Intermediate
Pasilla Bajío	Green	Light purple	Sparse	1	Pendant	Purple	White	Exserted	Dentate
Serrano Criollo de Morelos	Green	Light purple	Dense	1	Erect	Pale blue	White	Exserted	Dentate
Piquillo	Green	Purple	Sparse	1	Intermediate	Pale blue	White	Same level	Enture

SC, stem color; NA, nodal anthocyanin; SP, stem pubescence; NFA, number of flowers per axil; FP, flower position; AC, anther color; FC, filament color; SE, stigma exsertion; CM, calyx margin.

**Table 4:** Phenotypic values in leaf and fruit qualitative traits of the landraces and the reference varieties.

Varietal type/local name	LC	LS	AS	FCI	FCM	FS	FSBE	PP	FWC
Pebrera blanca	Green	Deltoid (17%) Ovate (83%)	Absent (33%) Present (67%)	Light green	Red	Triangular (50%) Blocky (50%)	Sunken	Intermediate (17%) Persistent (83%)	Consistent
Citró de Matances	Green (89%) Dark green (11%)	Deltoid (11%) Ovate (78%) Lanceolate (11%)	Absent (67%) Present (33%)	Green	Red (44%) Dark red (56%)	Elongate (33%) Triangular (44%) Blocky (22%)	Pointed (22%) Blunt (11%) Sunken (67%)	Intermediate (78%) Persistent (22%)	Consistent
Banya de cabra	Green	Ovate	Absent (67%) Present (33%)	Green	Red (33%) Dark red (67%)	Elongate (67%) Triangular (33%)	Pointed (25%) Sunken (75%)	Persistent	Consistent
Blau	Green (67%) Dark green (33%)	Ovate	Absent (67%) Present (33%)	Green	Dark red	Triangular		Intermediate	Consistent
Cirereta	Green	Ovate	Absent	Green	Dark red	Elongate	Pointed	Intermediate	Consistent
D’Envinagrar	Light green (50%) Green (50%)	Ovate	Absent	Light green	Light red	Elongate	Pointed	Intermediate	Consistent
Fulla d’Olivera	Dark green	Ovate (33%) Lanceolate (67%)	Absent	Light green (67%) Green (33%)	Red (67%) Dark red (33%)	Elongate	Pointed	Intermediate	Consistent
Ros	Light green (33%) Green (67%)	Ovate	Absent (33%) Present (67%)	Light green	Red	Triangular (33%) Blocky (67%)	Sunken	Intermediate (33%) Persistent (67%)	Consistent
Ros gruixat	Green	Ovate	Present	Light green	Red	Triangular	Pointed (20%) Sunken (80%)	Intermediate (25%) Persistent (75%)	Consistent
Ros prim	Light green (50%) Green (50%)	Ovate	Present	Light green	Red (50%) Dark red (50%)	Triangular	Sunken	Intermediate	Consistent
Tap de cortí	Light green (20%) Green (60%) Dark green (20%)	Ovate	Absent (60%) Present (40%)	Light green (25%) Green (75%)	Dark red	Triangular	Sunken	Intermediate	Consistent
California Wonder	Light green	Deltoid	Absent	Light green	Lemon-yellow	Blocky	Sunken	Persistent	Consistent
Chile Serrano	Dark green	Lanceolate	Absent	Dark green	Red	Elongate	Blunt	Slight	Consistent
Bola	Green	Ovate	Absent	Green	Red	Blocky	Blunt	Persistent	Consistent
Pasilla Bajío	Green	Lanceolate	Absent	Dark green	Brown	Elongate	Pointed	Intermediate	Consistent
Serrano Criollo de Morelos	Green	Lanceolate	Absent	Light green	Red	Triangular	Blunt	Intermediate	Consistent
Piquillo	Green	Ovate	Absent	Green	Dark red	Triangular	Sunken	Persistent	Consistent

LC, leaf color; LS, leaf surface; AS, anthocyanin spot; FCI, fruit color immature; FCM, fruit color mature; FS, fruit shape; FSBE, fruit shape at blossom end; PP, pedicel persistence; FWC, fruit wall consistency.

However, for most qualitative traits, variation was found within and between varietal types. Thus, there were traits where variation was found in both groups, the non-insular varieties and the Balearic peppers. In this regard, there were traits like nodal anthocyanin, leaf color, leaf shape, flower position, anther color, stigma exsertion, calyx margin, or fruit shape, where the ranges of variation observed in the Balearic landraces were similar to those found in the reference group (**Tables 3**, **4**). Some qualitative variation was found in the Balearic peppers in traits like the number of flowers per axil, filament color, or anthocyanin spots, where the control varieties were found monomorphic. These findings indicate that variation can be found in many traits like leaf shape, flower position, or anther color, even in a genetic background of a few varietal types native to these islands, in the same level as that observed in a more variable group of non-insular varietal types.

**Figure 2:**
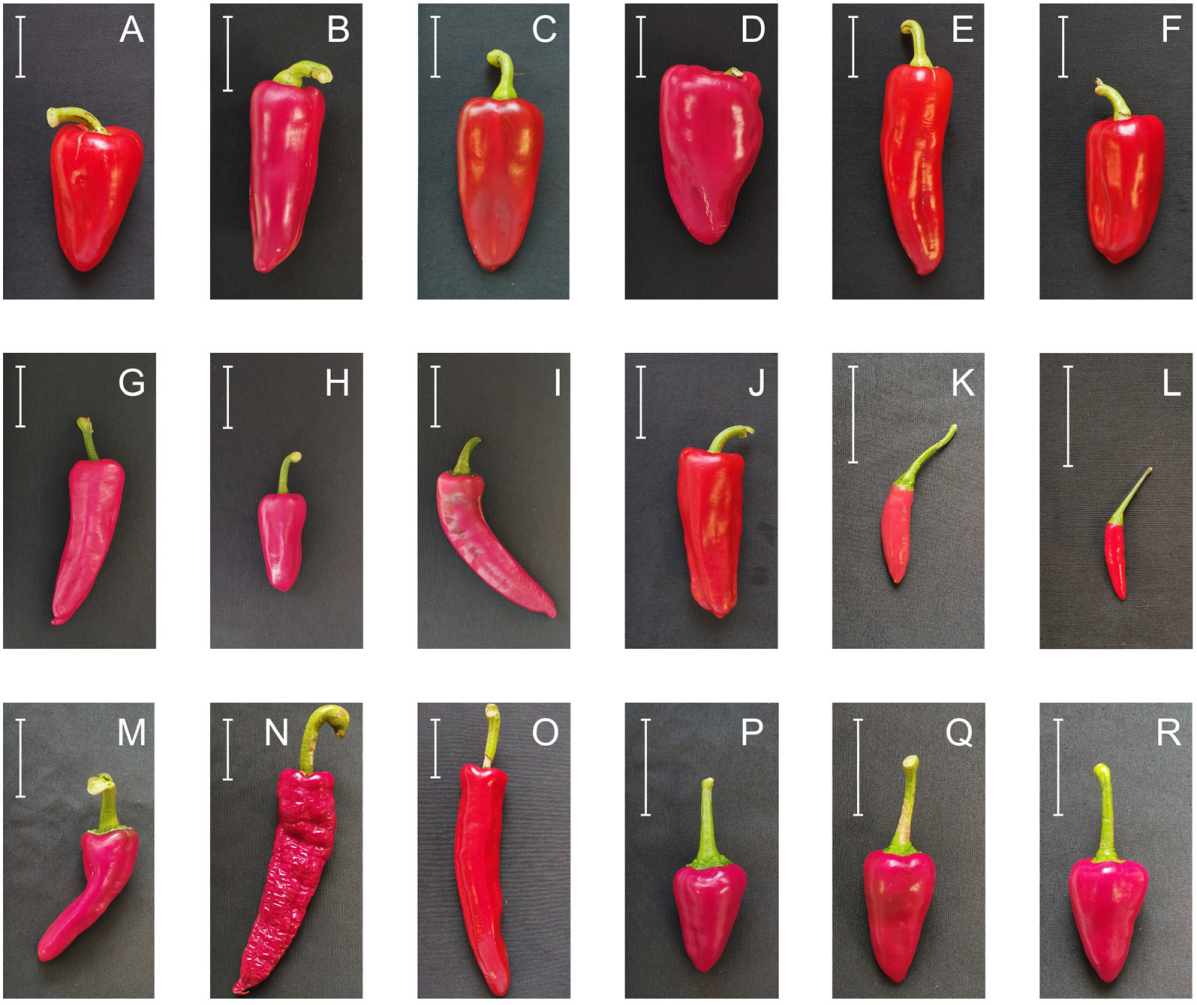
Examples of phenotypic variation in fruit morphology among accessions of Pebrera Blanca **(A–F)**, Citró de Matances **(G–L)**, Banya de Cabra **(M–O)**, and Tap de Cortí **(P–R)**. The white bar indicates 5 cm length.

Accessions from groups of Pebrera Blanca or Citró de Matances showed a considerable variation in traits like stigma exsertion or fruit shape (**Figure 2** **;** **Tables 3**, **4**). Also, Citró de Matances was relatively variable for stigma exsertion and fruit shape, Banya de Cabra for flower position and filament color, Blau for stigma exsertion, or Tap de Cortí for leaf color and stigma exsertion, taking into account the limitations in the number of accessions in the last three varietal groups (**Tables 3**, **4**). On the contrary, there were also cases of monomorphism or high uniformity within varietal types in other traits like fruit color at the intermediate stage or filament color.

In terms of flower morphology, reference varieties differed in stigma exsertion among them. Also, variation was found in stigma exsertion among reference varieties, as much as both among and within varietal types (**Tables 3**, **4**). Adaptation to the environment is a key factor for flower morphology (Yamada and Maki, 2014). Thus, flower trait variations among landraces could be due to adaptation to different ecological conditions. Finally, fruit color at the intermediate stage showed high variation between varietal types. For instance, unripped commercialized landraces such as Pebrera Blanca, Ros, Ros Prim, or Ros Gruixat showed the lightest color at the intermediate stage, indicating a historical farmer selection to improve the visual performance of these landraces.

#### 3.1.2 Quantitative traits

The quantitative traits analyzed showed a remarkable diversity. Moreover, diversity was found within and between all the studied varieties (**Table 5**; **Supplementary Table S2**). Monomorphic traits were few, and only the number of locules and fruit shape at blossom end showed very little variation among the studied varietal types. On the contrary, high variability was found within varietal types (**Supplementary Table S2**). As was observed for qualitative traits, some quantitative traits such as pollen morphological and biological viability and seed weight had similar ranges of variation when comparing Balearic landraces and the control group. Also, seed weight was found quite discriminating to discriminate among varietal types, in particular, the Balearic collection. Thus, most reference varieties ranged between 0.60 and 0.80 g/100 seeds, with the only exception of Pasilla Bajío, which achieved 1.29 g/100 seeds (**Table 5**). The means of the Balearic types ranged between 0.40 and 0.50 g/100 seeds for Blau, Cirereta, D’Envinagrar, and Fulla d’Olivera and 0.70 g/100 seeds of Pebrera Blanca and Banya de Cabra, and even a remarkable intravarietal variation was found in Citró de Matances (0.28–0.99 g/100 seeds) (**Table 5**; **Supplementary Table S2**).

**Table 5:** Mean values (mean ± standard deviation) in the studied quantitative traits of the landraces and the reference varieties.

Varietal type/local name	SL	FL	FA	FB	FLE	FWI	FW	NL	PMV	PBV	SW
Pebrera Blanca	166 ± 87	35.29 ± 1.9	36.42 ± 4.1	21.99 ± 4	131 ± 17	54 ± 12	151.67 ± 40.8	3.2 ± 0.6	0.74 ± 0.1	0.74 ± 0.1	0.71 ± 0.1
Citró de Matances	325 ± 15	35.88 ± 3.5	37.66 ± 4.7	19.25 ± 4.6	87 ± 40	2.5 ± 11	24.92 ± 19.7	2.42 ± 0.6	0.74 ± 0.1	0.68 ± 0.2	0.61 ± 0.2
Banya de Cabra	252 ± 164	33.58 ± 2.9	34.12 ± 4	16.3 ± 3.5	154 ± 46	35 ± 5	53.02 ± 23.6	3.27 ± 0.5	0.82 ± 0.1	0.82 ± 0.1	0.72 ± 0.1
Blau	372 ± 24	NA	NA	NA	NA	NA	NA	NA	0.61 ± 0.4	0.61 ± 0.4	0.52
Cirereta	255	35.1 ± 1.7	40.16 ± 1.4	18.06 ± 1.5	102 ± 20	17 ± 4	12.79 ± 5.6	2.2 ± 0.4	0.72	0.72	0.42
D’Envinagrar	495 ± 17	42.73 ± 2.1	42.46 ± 3.3	31.79 ± 5.8	86 ± 9	22 ± 3	13.79 ± 2.2	2.7 ± 0.5	0.78	0.78	0.5 ± 0.1
Fulla d’Olivera	212 ± 16	36.22 ± 3.9	39.62 ± 2.1	18.82 ± 3.6	102 ± 21	15 ± 3	10.68 ± 5.8	2.4 ± 0.5	0.81 ± 0.1	0.8 ± 0.1	0.48 ± 0.1
Ros	160 ± 66	35.5 ± 1.8	38.22 ± 3.8	20.53 ± 3.7	126 ± 28	46 ± 6	104.28 ± 31.6	3.27 ± 0.7	0.86 ± 0.1	0.86 ± 0.1	0.63
Ros Gruixat	225 ± 52	36.5 ± 3	38.61 ± 4.2	22.7 ± 5.1	144 ± 18	44 ± 5	114.65 ± 25.1	2.5 ± 0.5	0.68 ± 0.1	0.68 ± 0.1	0.63 ± 0.1
Ros Prim	218 ± 4	35.75 ± 2.6	39.43 ± 2.7	22.54 ± 3.5	142 ± 21	46 ± 4	118.07 ± 21.9	2.9 ± 0.3	0.5 ± 0.1	0.49 ± 0.1	0.65 ± 0.1
Tap de Cortí	184 ± 20	33.3 ± 1.8	32.48 ± 2.3	14 ± 1.9	61 ± 6	28 ± 2	21.81 ± 3.7	2.55 ± 0.5	0.81 ± 0.1	0.81 ± 0.1	0.55 ± 0.1
California Wonder	245	59.12 ± 3.3	−0.08 ± 2.5	56.24 ± 3	72 ± 4	62 ± 4	93.15 ± 8.1	3	0.07	0.05	0.78
Chile Serrano	85	41.93 ± 2.4	40.5 ± 1.1	26.95 ± 4	36 ± 4	13 ± 1	3.24 ± 1	2.4 ± 0.5	0.79	0.78	0.6
Bola	435	36.5 ± 1.3	35.59 ± 2.7	18.86 ± 1.5	33 ± 2	27 ± 3	14.61 ± 5.4	2.6 ± 0.5	0.39	0.26	0.7
Pasilla Bajío	485	28.03 ± 0.8	3.12 ± 1.1	3.73 ± 0.8	155 ± 12	29 ± 2	27.06 ± 7.7	2.2 ± 0.4	0.68	0.66	1.29
Serrano Criollo de Morelos	23	39.9 ± 2.6	39.83 ± 1.7	23.65 ± 2.4	101 ± 9	2 ± 1	16.52 ± 4.3	2.2 ± 0.4	0.88	0.83	0.74
Piquillo	25	36.13 ± 1.9	34.63 ± 3.5	17.2 ± 2.9	71 ± 9	41 ± 3	41.8 ± 9.3	2.6 ± 0.9	0.81	0.76	0.77

NA indicates missing data.

SL, stem length (mm); FL, fruit color lightness; FA, fruit color green/red; FB, fruit color blue/yellow; FLE, fruit length (mm); FWI, fruit width (mm); FW, fruit weight (g); NL, number of locules; PMV, pollen morphological viability (%); PBV, pollen biological viability (%); SW, seed weight (g).

As mentioned, a relevant variation between varietal groups was observed for fruit traits such as fruit length, width, fruit color at the mature stage, and fruit weight. Regarding fruit morphology, bigger fruits were observed in those landraces whose fruits are marketed at the unripe stage (Pebrera Blanca, Ros, Ros Prim, and Ros Gruixat), while the smallest fruits corresponded to those landraces whose fruits are used at the ripe stage (Cirereta, D’Envinagrar, or Fulla d’Olivera) (**Table 5**).

As observed for qualitative traits, Pebrera Blanca and Citró de Matances showed great variation for quantitative traits, while Mallorca landraces showed less variability within varietal types, taking into account variation limitations due to a small number of accessions of some Mallorca landraces (**Figure 2**; **Supplementary Table S2**). This wide variation between and within insular varieties has also been found in other collections such as the Ramellet tomato (*Solanum lycopersicum*) accessions in the Balearic Islands, with great diversity especially for leaf and fruit morphology (Bota et al., 2014). Bota et al. (2014) also observed significant differences between Ramellet accessions and a group of external accessions in tomato. Moreover, barley collections from the Canary Islands have also presented great variation among accessions within and among islands and a considerable diversification from continental accessions (Hagenblad et al., 2019).

Pollen viability was very variable among the reference varieties, ranging from very low values in the California Wonder breeding line (7% morphological, 5% biological) to very high in the Serrano types and Piquillo (≥80%) (**Table 5**; **Supplementary Table S2**), in correspondence to their varietal diversity and adaptation to different environments. A similar pattern was found among the Balearic accessions and varietal types, with means comprised between Ros Prim (approximately 50%) and Ros (86%), appearing extreme values in accessions like Ros-X6 (94%) or Blau-P50 (16%) (**Supplementary Table S2**).

#### 3.1.3 Principal component analysis

**Figure 3:**
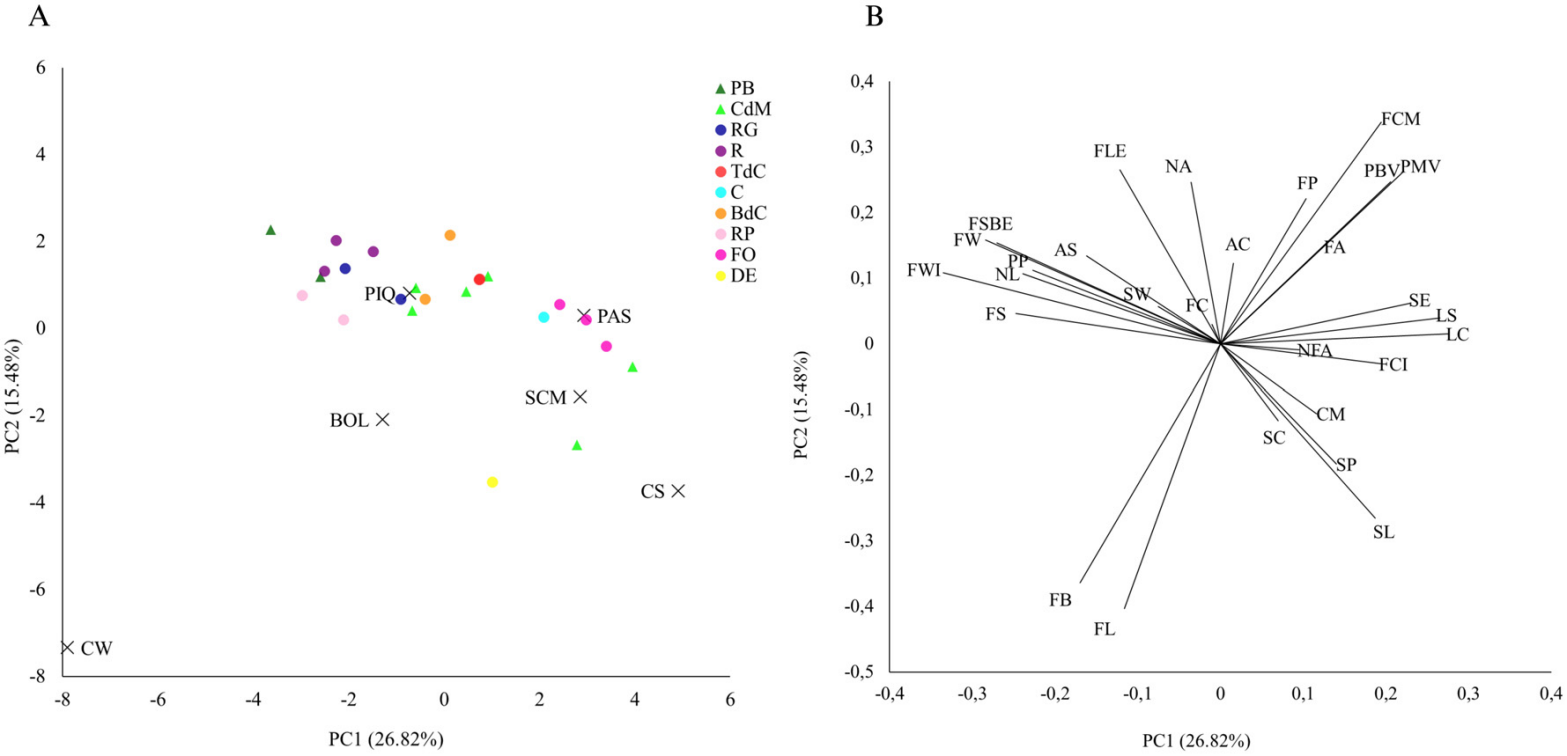
Principal component analysis using phenotypic data. **(A)** First (PC1) and second (PC2) principal components representing the selected accessions and the reference varieties and **(B)** the loading plot of PC1 and PC2.

The first two PCA components contributed to explain 42.30% of the observed variation, 26.82% by PC1 and 15.48% by PC2 (**Figure 3A**). According to this distribution, traits like fruit width, fruit shape, number of locules, leaf color, and leaf shape, among others, appeared as the most discriminant in terms of PC1 (i.e., the ones which appear in the extremes of PC1), while fruit color, and nodal anthocyanin in the stem were the more discriminant for PC2 (**Figure 3B**). Therefore, these traits seem to be the determinants to differentiate between accessions. In comparison to other reports, like those from Bota et al. (2014) in tomatoes or Pereira-Dias et al. (2020) in peppers, our findings are quite similar, confirming that these kinds of traits are usually the most useful to characterize accessions and discrimination among *Solanaceae* genotypes.

The first and second principal components, obtained with all the phenotypic data, enabled us to differentiate among the reference varieties, which appeared clearly separated among them and widely distributed in the PCA, mainly based on fruit descriptors such as fruit length, fruit width, and fruit weight (**Figure 3**). Fruit color at the fully ripe stage was key to differentiate California Wonder, i.e., yellow color, against the rest of them (**Figure 3B**). Regarding the Balearic landraces, Citró de Matances showed two clusters of accessions in the PCA, suggesting intravarietal variability. Thus, in general, Pebrera Blanca and all the Mallorca varietal types also differed among them and distributed widely in the PCA with some overlapping, but their intravarietal variation was smaller than that of the Citró de Matances, appearing quite close in the PCA. In this regard, we found that Citró de Matances, D’Envinagrar, and Banya de Cabra, as well as Ros and Ros Gruixat varietal types, partly overlapped, despite appearing on specific areas of the PCA (**Figure 3A**). Moreover, Pebrera Blanca or Ros types, used for their unripe and bigger-sized peppers were located in the upper left part of the PCA, separated from the rest of the landraces grown for their smaller fruits, mainly used fully ripe (**Figure 3A**).

#### 3.1.4 Intertrait correlations

**Figure 4:**
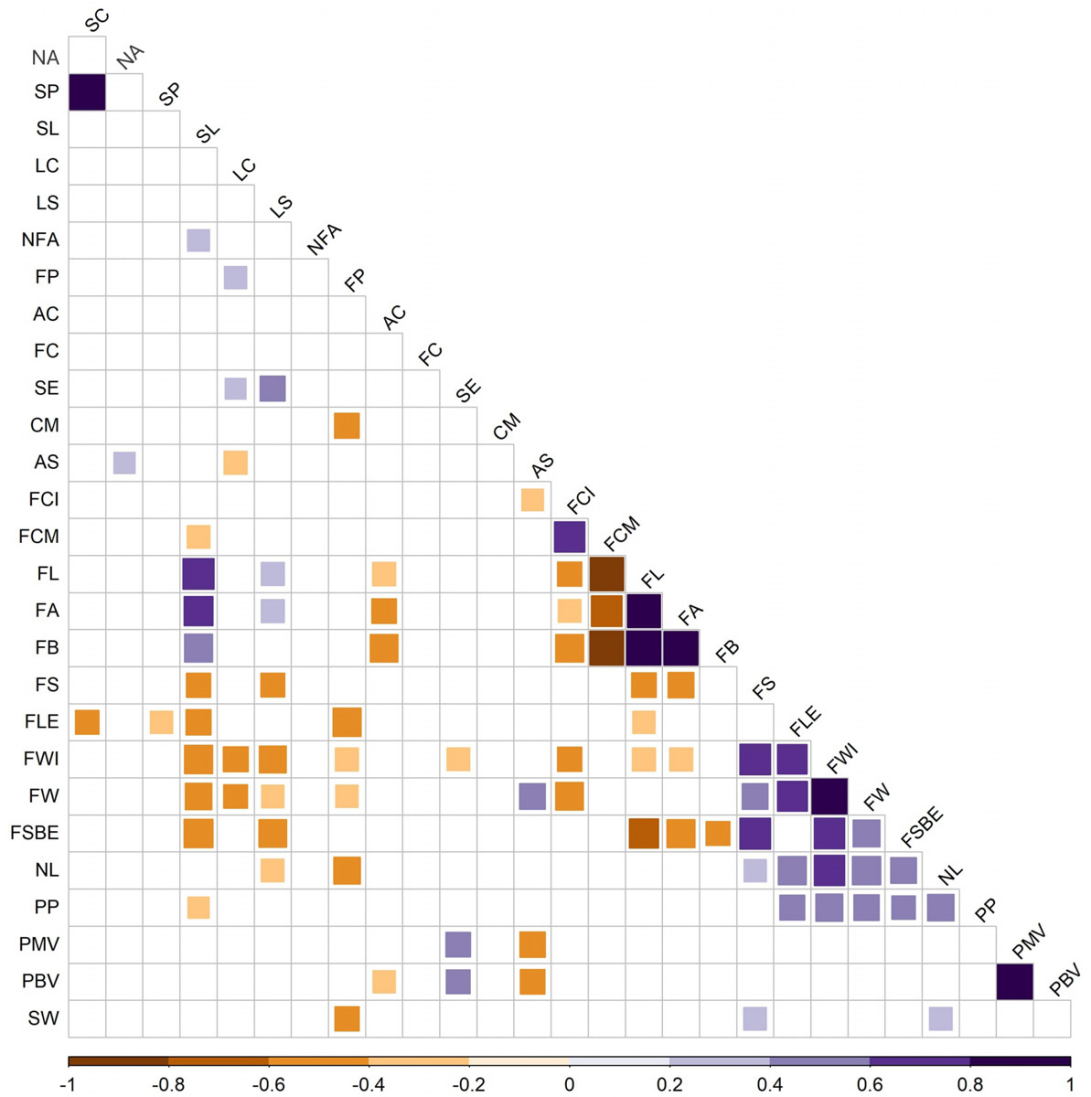
Intertrait correlations (Pearson coefficient *p* < 0.05) among plant (6), flower (6), fruit (14), pollen (2), and seed (1) traits phenotyped from 47 peppers accessions. Positive values are indicated in blue (increasing in value with darkness, i.e., from pale to dark blue). Negative values are indicated in orange (decreasing in value with darkness, i.e., from pale orange to dark orange/brown). SC, stem color; NA, nodal anthocyanin; SP, stem pubescence; SL, stem length; LC, leaf color; LS, leaf shape; NFA, number of flowers per axil; FP, flower position; AC, anther color; FC, filament color; SE, stigma exsertion; CM, calyx margin; AS, anthocyanin spot; FCI, fruit color immature stage; FCM, fruit color mature stage; FL, fruit brightness; FA, fruit color green/red; FB, fruit color blue/yellow; FS, fruit shape; FLE, fruit length; FWI, fruit width; FW, fruit weight; FSBE, fruit shape at blossom end; NL, number of locules; PP, pedicel persistence; PMV, pollen morphological viability; PBV, pollen biological viability.

Many correlations were found between traits with the phenotypic data of the studied populations (**Figure 4**). Positive correlations were found for plant descriptors such as stem color and stem pubescence, indicating that purple stems tend to show more density of the hairs. One negative correlation was found for flower descriptors, between flower position and calyx margin, indicating that a pendant flower position is related to a dentate calyx margin in the developing fruit. Morphological and biological pollen viabilities were highly positively correlated indicating that both parameters were estimating pollen grain viability in an equivalent way. A large number of correlations were found between fruit traits. For instance, darker fruits in the immature stage seemed to be related to the absence of anthocyanin spot and to darker fruits in the mature stage. Also, numerous positive correlations were observed for fruit shape; for example, larger fruits were related to wider fruits, indicating that, in the studied populations, landraces with bigger fruits tend to be more blocky-shaped fruits. Some correlations were found between different organ traits, e.g., longer stems and round leaves were correlated with smaller (lower length, width, and weight) and elongated fruits. Moreover, lower pollen viabilities were correlated with the presence of anthocyanin spots in the fruit and an inserted stigma in the flower. Finally, a higher seed weight seemed to be related to a pendant flower, an elongated, blocky-shaped fruit with numerous locules.

### 3.2 Genotyping-by-sequencing characterization

#### 3.2.1 SNP calling and sequencing

The sequencing of the Balearic and reference accessions produced over 90 million raw paired-end reads and approximately 10 Gb of data. After demultiplexing, cleaning, and trimming, selected reads were aligned to the pepper genome CM334 (Serrano Criollo de Morelos version 1.55) (Kim et al., 2014). In total, 1,197,532 polymorphic sites, distributed over the 12 chromosomes, were retained after SNP calling and basic filtering, and 9,508 high-quality SNPs were selected for diversity analysis as described in Section 2.4.

Heterozygosity was calculated using the 1,197,532 SNPs retained after basic filters. Heterozygosity was comprised between 0.38% and 1.95% in the Balearic collection (**Table 6**, **Supplementary Table S3**). Low heterozygosity values are usually expected for autogamous species, despite being exposed to open pollination like landraces, as has been observed for other autogamous species such as tomato (Bota et al., 2014). In pepper, also an autogamous species, low values for heterozygosity have previously been reported. For instance, Taranto et al. (2016) described mean heterozygosity values of 2.4% in a collection of *C. annuum* with different origins. Similarly, Pereira-Dias et al. (2019) found heterozygosity mean values of 2.97% for a collection of *C. annum* varieties. Even more, higher mean heterozygosity values for other pepper populations have been obtained when including wild-related species (Ibiza et al., 2012; Cheng et al., 2016; Lee et al., 2016), which are exposed to conditions where allogamy is favored.

**Table 6:** Heterozygosity mean values (Het, %) per varietal types and heterozygosity range of values among the accessions (minimum–maximum) within each varietal type.

Varietal type/local name	Het	Range
*Pebrera Blanca*	0.80	[0.55–1.14]
*Citró de Matances*	0.65	[0.38–1.11]
*Banya de Cabra*	0.88	[0.72–1.03]
*Blau*	0.80	[0.77–0.84]
*Cirereta*	0.82	–
*D’Envinagrar*	1.95	–
*Fulla d’Olivera*	0.77	[0.69–0.86]
*Ros*	0.74	[0.74–0.75]
*Ros Gruixat*	0.81	[0.75–0.90]
*Ros Prim*	0.74	[0.72–0.76]
*Tap de Cortí*	0.71	[0.56–0.92]

Regarding Eivissa accessions, Pebrera Blanca showed slightly higher heterozygosity values than Citró de Matances, with a mean value of 0.80% in the former and a mean value of 0.65% in the latter (**Table 6**). For Mallorca varietal types and accessions, heterozygosity values varied a little bit more in a general view, maybe due to differences in the number of accessions genotyped for the Mallorca landraces (**Table 6**, **Supplementary Table S3**).

#### 3.2.2 Principal component analysis

**Figure 5:**
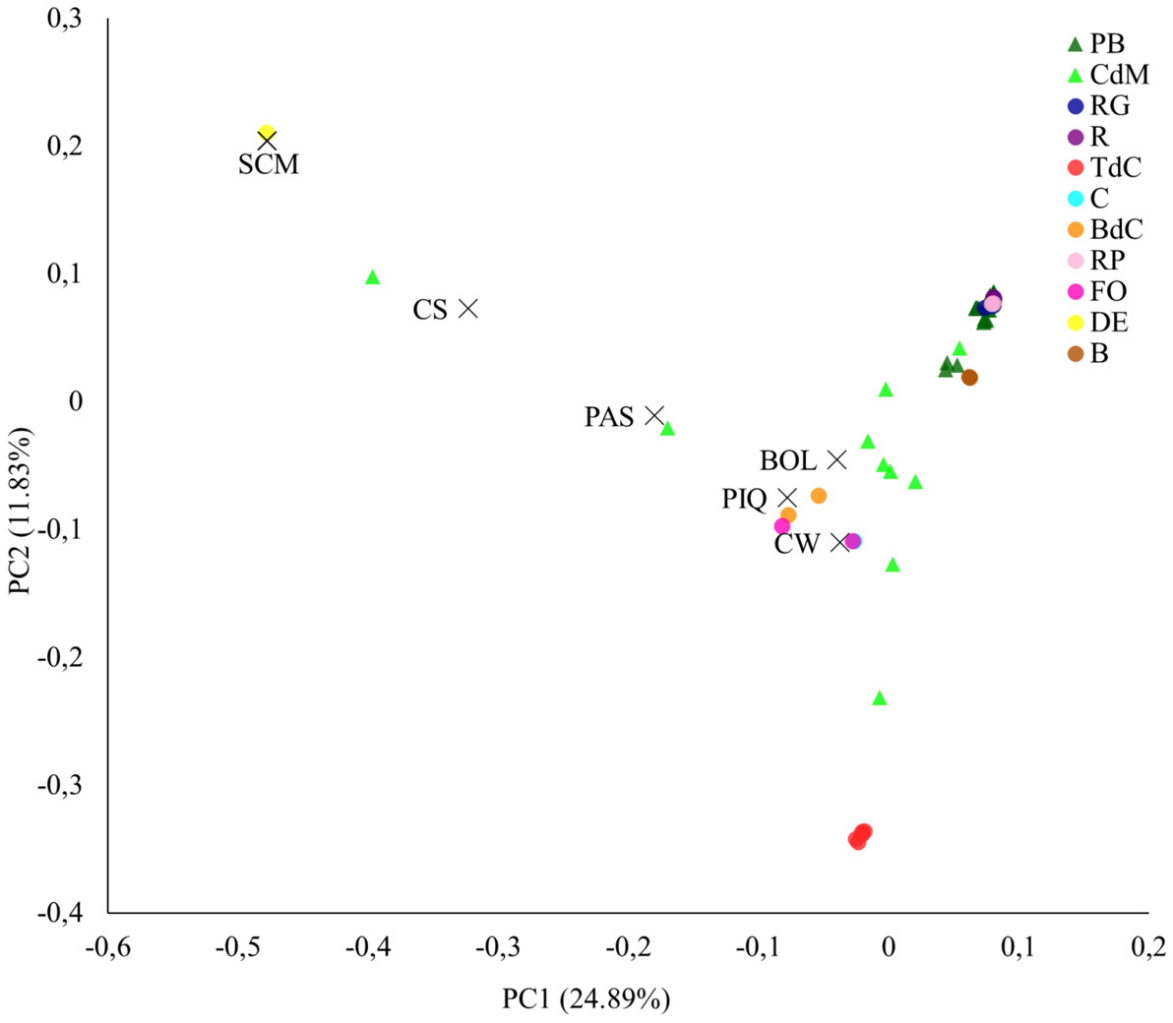
Principal component analysis. First (PC1) and second (PC2) principal components representing the Balearic accessions and the reference varieties, based on the 9,508 filtered SNPs.

The PCA provided interesting results about the studied varietal groups. The reference varieties, as expected for their genetic divergence, appeared widely distributed throughout the PCA, with California Wonder, Bola, and Piquillo in the central area (i.e., PC1 = 0, PC2 = 0) and Chile Serrano, Pasilla Bajío, and, particularly, Serrano Criollo de Morelos, in the most extreme positions, and clearly separated from the main cloud of accessions (**Figure 5**). Regarding the Balearic materials, the Citró de Matances accessions covered a wide distribution in the PCA, with several accessions located in the main cloud but others appearing in far positions close to Chile Serrano, Bola, and Pasilla Bajío (**Figure 5**). These findings are in agreement with the phenotyping intravarietal variation found within this type (**Supplementary Table S2**) and confirm that, on occasions, one local name may encompass a range of phenotypes as well as different genetic strains, as reported by Pereira-Dias et al. (2020) in peppers, Nuijten and Almekinders (2008) in Baras rice in Gambia, or Mar and Holly (2000) in beans in Hungary, whose common names refer to just one main morphological or agronomical trait but actually encompass a plethora of genotypes and morphologies for other traits.

Finally, in the case of Pebrera Blanca accessions, they appeared in the corner of positive values of PC1 and PC2 although they seem to be divided into two groups slightly separated, suggesting two differentiated genetic subpopulations with the available accessions. These results agree with the diversity found for phenotypic data and fruit morphology for this varietal type (**Tables 3**, **4**).

#### 3.2.3 Phylogenetic tree and kinship matrix

**Figure 6:**
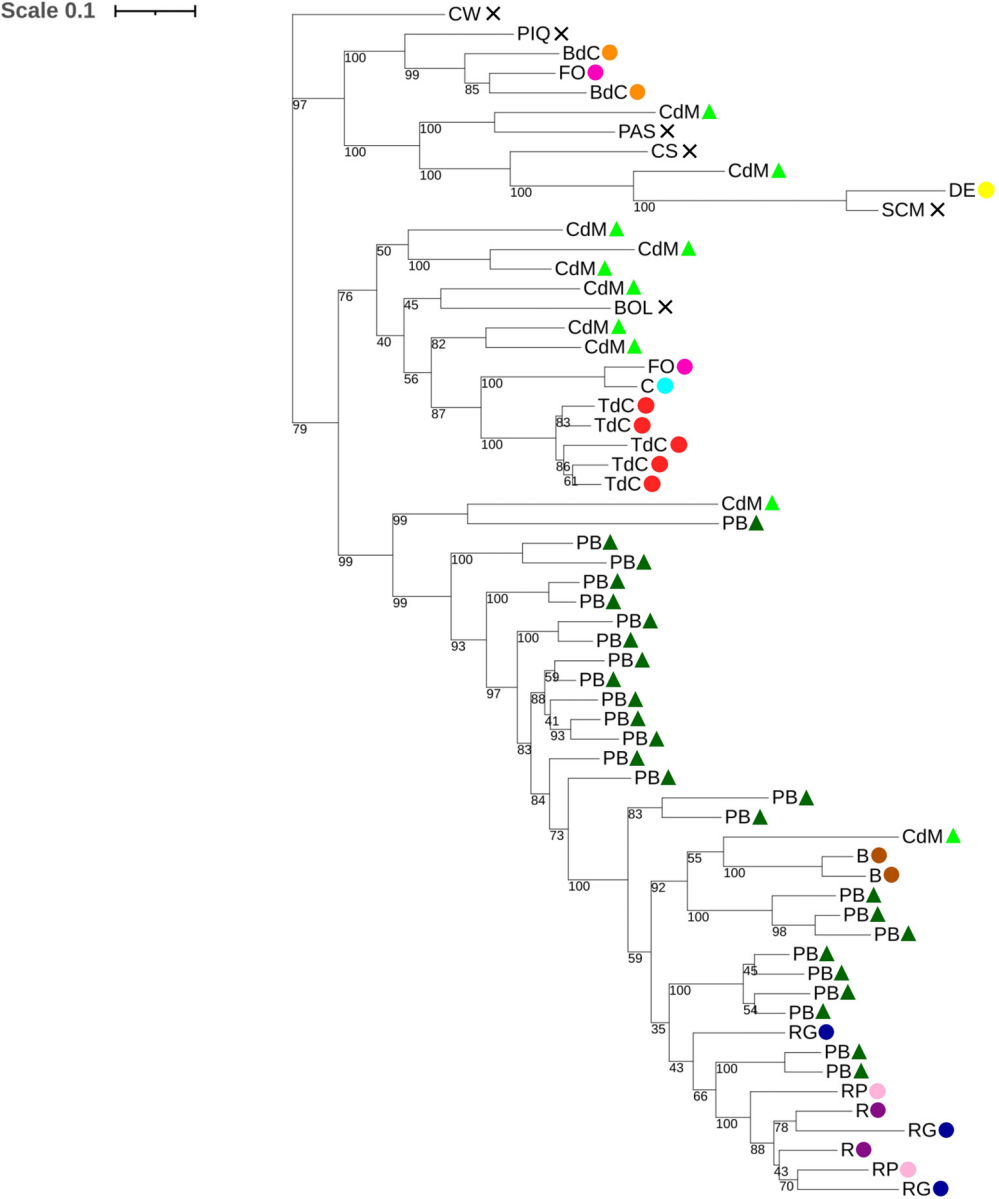
Dendrogram created from 1,000 bootstrap replicates for the studied Balearic accessions and the reference varieties, based on the 9,508 filtered SNPs.

The dendrogram showed accessions clustered by varietal group, but not clearly clustered by origin, indicating the existence of interisland crosses or admixture due to the interchange of seeds between Mallorca and Eivissa farmers (**Figure 6**). However, some landraces were clustered together and clearly differentiated from the others like Tap de Cortí or Blau, indicating a unique genetic pattern that will be further discussed in the admixture section (Section 3.2.4). On the other hand, some varietal types like Fulla d’Olivera and Cirereta appeared in the same dendrogram branch, which could be explained because both landraces show similar fruit shapes and are usually used as a spice, and this may be related to the same genetic basis. Interestingly, cultivars usually harvested and sold at the unripe stage like Ros, Ros Gruixat, Ros Prim, and Pebrera Blanca, with bigger fruits, also clustered and were not differentiated clearly by varietal group, suggesting selection and genetic exchange in these materials. Moreover, as it has been seen in the PCA and in the phenotyping data, differentiated genetic lineages may be observed in Pebrera Blanca and Citró de Matances, with various genetic clusters of accessions each, one cluster per varietal type very close and some accessions mixed with other landraces (**Figures 5**, **6**). The group of reference varieties also appeared close in the dendrogram, with the only exception of Bola. However, some accessions of Banya de Cabra, Fulla d’Olivera, and Citró de Matances appeared clustered together with the reference varieties. This might suggest that, despite most Mallorca and Eivissa landraces had a different breeding process, some materials could conserve some genetic similarities with the controls or even had experienced some genetic exchange in the past.

**Figure 7:**
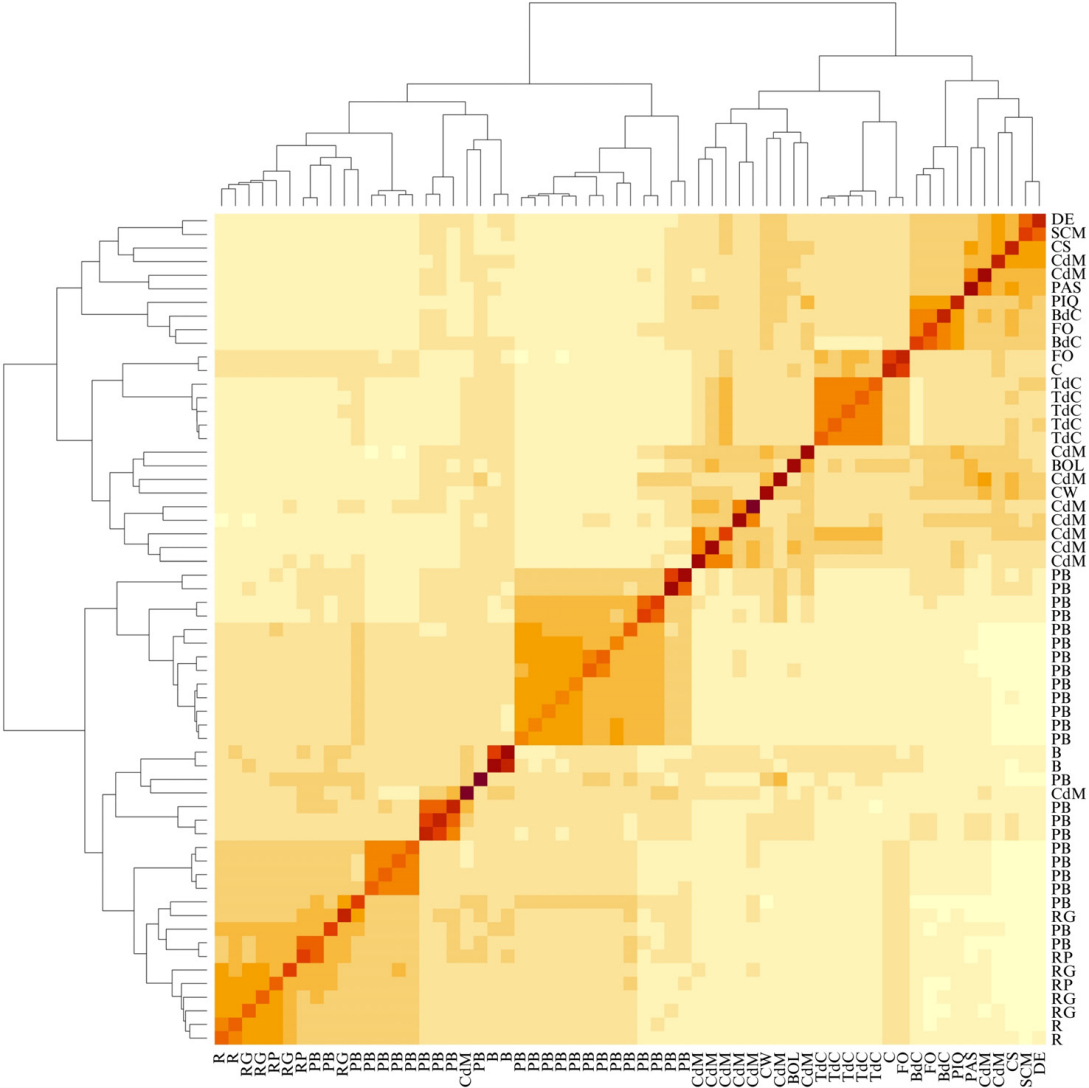
Heatmap of kinship matrix with the tree shown on the top and left of the studied Balearic accessions and the reference varieties, based on the 9,508 filtered SNPs.

The kinship matrix confirmed the data obtained in the dendrogram and the PCA. Thus, the reference controls were clustered and differentiated from Balearic landraces, with the only exception of some Citró de Matances accessions, whose high genetic intravarietal diversity, as observed in the PCA, explains such overlapping with the reference varieties (**Figures 5**, **7**). All Tap de Cortí accessions were clustered unequivocally, suggesting breeding and selection processes very close to this varietal type. Also, Banya de Cabra and Fulla d’Olivera were clustered together, suggesting little genetic differences between both landraces.

Fruit traits such as fruit morphology and color were key to differentiate cultivars, as it was found in both phenotypic and genotypic PCAs (**Figures 3A**, **5**). In this regard, landraces used for the unripe commercialized peppers Ros, Ros Gruixat, and Ros Prim were clustered together, indicating great genetic similarities. These results could be associated with quality traits in these unripe-used peppers due to historical farmer selection preferences and/or genetic exchange (**Figure 7**). As it has been previously mentioned, no clear separation was observed between Eivissa and Mallorca blocks. Thus, despite considering that some genotypes include few accessions, we cannot contemplate the occurrence of different genetic backgrounds among both islands with the available information.

#### 3.2.4 Admixture analysis

**Figure 8:**
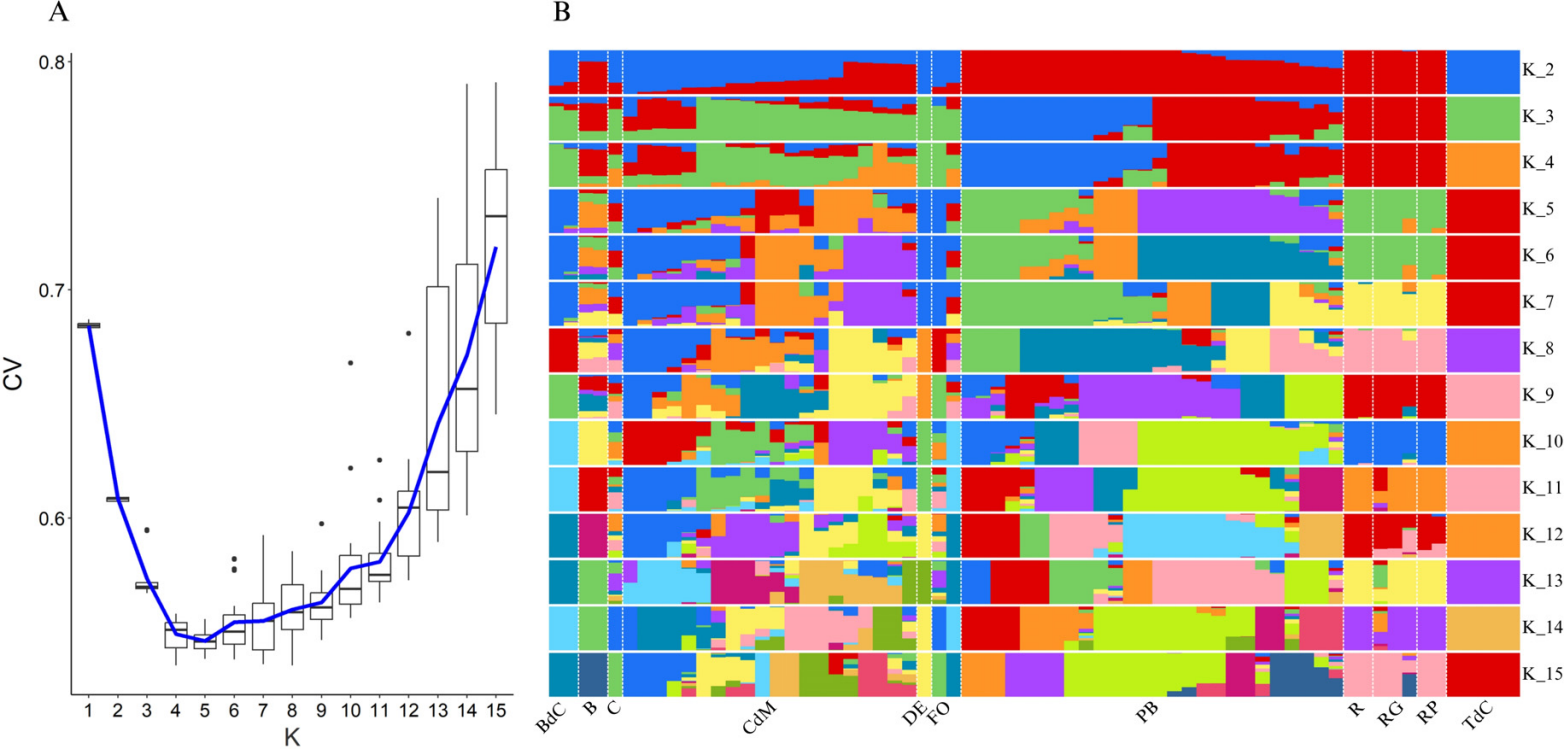
Admixture population structure analysis using the filtered SNPs of Mallorca and Eivissa landraces. **(A)** Cross-validation (CV) error plot to estimate the best fitting *K* value and **(B)** structuration of the population according to the number of clusters *K* (2–15). Colors represent different assigned clusters.

For further elucidation of the genetic structure of the collection of landraces, an admixture analysis was performed (**Figure 8**). The estimated cross-validation (CV) error (**Figure 8A**) indicated that *K* = 5 was the best model for the analysis of the studied collection. This means that a model based on five genetic clusters provides the most accurate explanation for the genetic variation observed in our study.

In this regard and considering the *K* = 5 model, the admixture analysis confirmed the findings of the PCA (**Figure 5**) and provided some more details of interest (**Figure 8B**). Thus, some landraces such as Tap de Cortí, Banya de Cabra, and D’Envinagrar showed genetic patterns without admixture. Moreover, the group of Ros accessions, i.e., Ros, Ros Gruixat, and Ros Prim, also showed a unique genetic background (green) with the only exception of a very little occurrence (<10%) of an additional background (orange), and the same was found in Banya de Cabra, mainly blue background with very few of other backgrounds (**Figure 8B**). Such findings suggest that Ros, Ros Gruixat, and Ros Prim might share a common origin despite their seeds being obtained from different sources or locations. In short, they may have some morphological differences, but this is barely reflected in their genome.

On the contrary, other varietal types like Blau, Cirereta, or Fulla d’Olivera showed more complex patterns, with a mixture of genetic backgrounds (**Figure 8B**). This fact was even more remarkable in the case of the Pebrera Blanca and, particularly, Citró de Matances varietal groups. Nevertheless, it is necessary to take into account the possible existence of differences due to the different number of accessions available on-farm to represent each varietal group.

In the case of the Pebrera Blanca population, the admixture analyses showed two clearly different lines, one which could be considered almost a unique background (purple) with a limited presence of other minor backgrounds (<10%) and another which appears as a mixture of two main backgrounds (green and orange) (**Figure 8B**). These results are in agreement with the PCA based on the SNP polymorphisms (**Figure 5**), confirming the presence of two subpopulations of this varietal type. Finally, also in agreement with the PCA, the Citró de Matances accessions encompassed a plethora of genetic patterns, which confirms the intravarietal heterogeneity of this varietal group observed at both the phenotypic and genetic levels (**Tables 3**–**5**; **Figures 3A**, **5**). This high diversity found in landraces like Citró de Matances and Pebrera Blanca could determine the necessary efforts for typification and to protect these landraces as relevant representatives of agrodiversity in the islands. In this regard, the possibility of new allele combinations during *in-situ* conservation needs to be taken into account, mainly due to gene exchange if grown open field together with other varieties, as often occurs in these agrifood systems. On the contrary, varieties with low diversity (and therefore clearly defined in phenomic terms) like Tap de Cortí could be conserved easier by farmers and directly used for breeding purposes and gene introgressions.

## 4 Concluding remarks

Our study on endemic peppers representative of the Balearic Islands has revealed a very diverse picture in terms of morphological variation and genetic structure. The on-farm cultivation and multiplication of these landraces has driven different genetic lineages, not always necessarily toward heterogeneous varieties as pepper is considered an autogamous crop. Thus, varietal types like Citró de Matances from Eivissa showed a plethora of morphologies and genetic backgrounds among their accessions, as well as a range of heterozygosity values (0.38%–1.11%) in comparison to other varietal types, revealing that under a common term, many lineages can be encompassed in local materials. In other cases, varietal types like Tap de Cortí or the Ros group were quite uniform in morphologies and presented a unique genetic background, with low levels of heterozygosity (<0.90%). Finally, other types like Pebrera Blanca represent intermediate cases, where two genetic subpopulations or lineages appeared, in agreement with slight morphological differences as can be observed in their rectangular elongated fruits (e.g., some accessions with fruits a bit more elongated vs. some others a bit shorter). These results suggest, on the whole, a diverse genetic structure, probably due to differences in farmers’ practices during multiplication and breeding, or cross-pollination levels. This genetic and phenotypic diversity could be of great interest for future breeding projects to improve resilience in commercial peppers by broadening the genetic background of the breeding materials. Finally, farmers could also benefit from the information obtained in this work, which can be used for developing genetic profiles for each landrace, determining a more efficient on-farm and *ex-situ* conservation and speeding up the selection process. Moreover, this work could be used for future typification and legal protection of these local materials for scaling up toward more commercial exploitations.

## Data Availability

The original raw data presented in the study are publicly available. This data can be found here: https://www.ncbi.nlm.nih.gov/bioproject/PRJNA1173395.
