# Supplementary material for: Genetic diversity, population structure, and phylogeny of insular Spanish pepper landraces (Capsicum annuum L.) through phenotyping and genotyping-by-sequencing
Source: Front Plant Sci. 2024 Oct 30;15:1435427. doi: 10.3389/fpls.2024.1435427 (PMC11557316; doi:10.3389/fpls.2024.1435427)
Supplement: Supplementary file 1 [file Table1.docx]

**Supplementary Table 1.** List of studied accessions with corresponding code, genebank code, varietal type, and origin.

| Accession | Genebank code | Varietal type | Origin |  | Accession | Genebank code | Varietal type | Origin |
| --- | --- | --- | --- | --- | --- | --- | --- | --- |
| P1 | PI-0152 | Pebrera Blanca | Ibiza |  | P35 | PI-0014 | Citró de Matances | Ibiza |
| P2 | PI-0153 | Pebrera Blanca | Ibiza |  | P36 | PI-0071 | Citró de Matances | Ibiza |
| P3 | PI-0155 | Pebrera Blanca | Ibiza |  | P37 | PI-0069 | Citró de Matances | Ibiza |
| P4 | PI-0156 | Pebrera Blanca | Ibiza |  | P38 | PI-0251 | Citró de Matances | Ibiza |
| P5 | PI-0157 | Pebrera Blanca | Ibiza |  | P39 | - | Citró de Matances | Ibiza |
| P6 | PI-0159 | Pebrera Blanca | Ibiza |  | P40 | BGHZ5244 | Citró de Matances | Ibiza |
| P7 | PI-0160 | Pebrera Blanca | Ibiza |  | P41 | BGHZ3337 | Citró de Matances | Ibiza |
| P8 | PI-0161 | Pebrera Blanca | Ibiza |  | P42 | BGHZ5245/1 | Citró de Matances | Ibiza |
| P9 | PI-0162 | Pebrera Blanca | Ibiza |  | P43 | PI-0010/5 | Citró de Matances | Ibiza |
| P10 | PI-0163 | Pebrera Blanca | Ibiza |  | P44 | PI-0257 | Citró de Matances | Ibiza |
| P11 | PI-0164 | Pebrera Blanca | Ibiza |  | P45 | PI-0256 | Citró de Matances | Ibiza |
| P12 | PI-0165 | Pebrera Blanca | Ibiza |  | P46 | PI-0258 | Citró de Matances | Ibiza |
| P13 | PI-0166 | Pebrera Blanca | Ibiza |  | P47 | PI-0073 | Citró de Matances | Ibiza |
| P14 | PI-0167 | Pebrera Blanca | Ibiza |  | P48 | BGIB069* | Banya de cabra | Mallorca |
| P15 | PI-0168 | Pebrera Blanca | Ibiza |  | P49 | BGIB086 | Banya de cabra | Mallorca |
| P16 | PI-0169 | Pebrera Blanca | Ibiza |  | P50 | BGIB085 | Blau | Mallorca |
| P17 | PI-0170 | Pebrera Blanca | Ibiza |  | P51 | BGIB166 | Blau | Mallorca |
| P18 | PI-0171 | Pebrera Blanca | Ibiza |  | P52 | BGIB229 | Cirereta | Mallorca |
| P19 | PI-0068 | Pebrera Blanca | Ibiza |  | P53 | BGIB137 | D'envinagrar | Mallorca |
| P20 | PI-0264 | Pebrera Blanca | Ibiza |  | P54 | BGIB068 | Fulla d'olivera | Mallorca |
| P21 | - | Pebrera Blanca | Ibiza |  | P55 | BGIB152 | Fulla d’olivera | Mallorca |
| P22 | PI-0253 | Pebrera Blanca | Ibiza |  | P56 | BGIB160 | Ros | Mallorca |
| P23 | PI-0265 | Pebrera Blanca | Ibiza |  | P57 | BGIB222* | Ros | Mallorca |
| P24 | PI-0263 | Pebrera Blanca | Ibiza |  | P58 | BGIB039 | Ros gruixat | Mallorca |
| P25 | PI-0250 | Pebrera Blanca | Ibiza |  | P59 | BGIB091 | Ros gruixat | Mallorca |
| P26 | PI-0249 | Pebrera Blanca | Ibiza |  | P60 | BGIB084* | Ros gruixat | Mallorca |
| P27 | PI-0015/3 | Citró de Matances | Ibiza |  | P61 | BGIB070 | Ros prim | Mallorca |
| P28 | PI0117/4A | Citró de Matances | Ibiza |  | P62 | BGIB087 | Ros prim | Mallorca |
| P29 | PI-0117/4B | Citró de Matances | Ibiza |  | P63 | BGIB195 | Tap de cortí | Mallorca |
| P30 | PI-0079A/2 | Citró de Matances | Ibiza |  | P64 | BGIB022* | Tap de cortí | Mallorca |
| P31 | PI-0208 | Citró de Matances | Ibiza |  | P65 | BGIB110* | Tap de cortí | Mallorca |
| P32 | - | Citró de Matances | Ibiza |  | P66 | BGIB128 | Tap de cortí | Mallorca |
| P33 | PI-0254 | Citró de Matances | Ibiza |  | P67 | BGIB136 | Tap de cortí | Mallorca |
| P34 | PI-0255 | Citró de Matances | Ibiza |  |  |  |  |  |
