# Supplementary material for: Genetic diversity, population structure, and phylogeny of insular Spanish pepper landraces (Capsicum annuum L.) through phenotyping and genotyping-by-sequencing
Source: Front Plant Sci. 2024 Oct 30;15:1435427. doi: 10.3389/fpls.2024.1435427 (PMC11557316; doi:10.3389/fpls.2024.1435427)
Supplement: Supplementary file 2 [file Table2.docx]

**Supplementary Table 2.** Phenotyping values (mean ± standard deviation, in traits where several measurements were possible) for the quantitative traits analysed in all the selected accessions and reference varieties. NA indicates missing data.

| **Accession** | **SL^1^** | **FL** | **FA** | **FB** | **FLE** | **FWI** | **FW** | **NL** | **PMV** | **PB)** | **SW** |
| --- | --- | --- | --- | --- | --- | --- | --- | --- | --- | --- | --- |
| ***Pebrera Blanca*** |  |  |  |  |  |  |  |  |  |  |  |
| P4 | 115 | NA | NA | NA | NA | NA | NA | NA | 80 | 80 | 0.82 |
| P6 | 5 | 34.72 ± 1.3 | 35.66 ± 2.6 | 19.89 ± 3.7 | 127 ± 8 | 57 ± 6 | 179.54 ± 37.4 | 3.2 ± 0.4 | 64 | 64 | 0.69 |
| P13 | 225 | NA | NA | NA | NA | NA | NA | NA | 72 | 72 | 0.64 |
| P23 | 27 | NA | NA | NA | NA | NA | NA | NA | NA | NA | 0.73 |
| P25 | 17 | 36.89 ± 1.6 | 39.92 ± 2.9 | 24.99 ± 3.7 | 147 ± 18 | 43 ± 3 | 113.32 ± 20.3 | 3 ± 0.7 | 67 | 67 | 0.62 |
| P26 | NA | 34.27 ± 1.7 | 33.68 ± 4 | 21.09 ± 2.9 | 118 ± 7 | 63 ± 12 | 162.13 ± 32.8 | 3.4 ± 0.5 | 85 | 85 | 0.76 |
|  |  |  |  |  |  |  |  |  |  |  |  |
| ***Citró de matances*** |  |  |  |  |  |  |  |  |  |  |  |
| P27 | 30 | 33.82 ± 1.9 | 35.43 ± 1.9 | 16.61 ± 2.4 | 110 ± 20 | 31 ± 2 | 32.1 ± 9.2 | 2.8 ± 0.8 | NA | NA | 0.84 |
| P29 | 195 | 34.5 ± 1.7 | 34.54 ± 2.8 | 17.06 ± 2.3 | 78 ± 10 | 2.98 ± 2 | 27.99 ± 4.4 | 2 | 76 | 75 | 0.65 |
| P32 | 235 | 32.83 ± 0.8 | 33.64 ± 3 | 14.89 ± 2.9 | 109 ± 27 | 3.38 ± 4 | 29.85 ± 13.9 | 2.2 ± 0.4 | 90 | 89 | 0.99 |
| P33 | 19 | 33.04 ± 0.7 | 33.82 ± 2 | 15.28 ± 2.4 | 137 ± 9 | 3.02 ± 2 | 46.51 ± 9.7 | 2.6 ± 0.5 | 62 | 45 | 0.69 |
| P34 | 22 | 34.23 ± 2.4 | 33.27 ± 3.1 | 16.8 ± 4.2 | 96 ± 8 | 3.6 ± 4 | 34.86 ± 15.1 | 2.6 ± 0.5 | 66 | 49 | 0.71 |
| P42 | 285 | 34.49 ± 2.6 | 39.12 ± 2.2 | 21.93 ± 2.8 | 132 ± 11 | 3.04 ± 5 | 45.3 ± 20.6 | 3.2 ± 0.4 | NA | NA | 0.46 |
| P44 | 555 | 38.99 ± 0.9 | 41.39 ± 1.5 | 21.17 ± 2.3 | 53 ± 3 | 1.54 ± 1 | 4.94 ± 0.5 | 2.2 ± 0.4 | 88 | 86 | 0.48 |
| P45 | 57 | 41.26 ± 3 | 44.5 ± 1.2 | 25.9 ± 2.8 | 46 ± 4 | 10 ± 1 | 2.13 ± 0.3 | 2 | NA | NA | 0.44 |
| P46 | 375 | 39.73 ± 1.2 | 43.24 ± 1.4 | 23.58 ± 2.5 | 19 ± 2 | 8 ± 1 | 0.63 ± 0.1 | 2.2 ± 0.4 | 61 | 61 | 0.28 |
|  |  |  |  |  |  |  |  |  |  |  |  |
| ***Banya de Cabra*** |  |  |  |  |  |  |  |  |  |  |  |
| X1 | 39 | 34.97 ± 2.3 | 37.37 ± 3.1 | 18.52 ± 3.2 | 107 ± 40 | 29 ± 4 | 30.85 ± 9 | 3.2 ± 0.4 | 78 | 78 | 0.69 |
| P48 | 7 | 31.88 ± 2.2 | 31.32 ± 2.7 | 14 ± 3 | 191 ± 19 | 37 ± 4 | 65.94 ± 24.9 | 3.4 ± 0.5 | NA | NA | 0.67 |
| P49 | 295 | 33.91 ± 3.3 | 33.66 ± 3.9 | 16.39 ± 3.1 | 163 ± 33 | 38 ± 4 | 62.26 ± 17.8 | 3.2 ± 0.4 | 86 | 86 | 0.79 |
|  |  |  |  |  |  |  |  |  |  |  |  |
| ***Blau*** |  |  |  |  |  |  |  |  |  |  |  |
| X2 | 38 | NA | NA | NA | NA | NA | NA | NA | 89 | 87 | NA |
| P50 | 39 | NA | NA | NA | NA | NA | NA | NA | 16 | 16 | NA |
| P51 | 345 | NA | NA | NA | NA | NA | NA | NA | 79 | 79 | 0.52 |
|  |  |  |  |  |  |  |  |  |  |  |  |
| ***Cirereta*** |  |  |  |  |  |  |  |  |  |  |  |
| P52 | 255 | 35.1 ± 1.7 | 40.16 ± 1.4 | 18.06 ± 1.5 | 102 ± 20 | 17 ± 4 | 12.79 ± 5.6 | 2.2 ± 0.4 | 72 | 72 | 0.42 |
|  |  |  |  |  |  |  |  |  |  |  |  |
| ***D’Envinagrar*** |  |  |  |  |  |  |  |  |  |  |  |
| X3 | 375 | 41.92 ± 1.9 | 39.93 ± 2.6 | 27.26 ± 3.6 | 79 ± 10 | 2.46 ± 2 | 14.82 ± 1.3 | 2.8 ± 0.4 | NA | NA | 0.42 |
| P53 | 615 | 43.54 ± 2 | 44.99 ± 1.4 | 36.32 ± 3.4 | 91 ± 3 | 1.94 ± 2 | 12.76 ± 2.5 | 2.6 ± 0.5 | 78 | 78 | 0.57 |
|  |  |  |  |  |  |  |  |  |  |  |  |
| ***Fulla d’Olivera*** |  |  |  |  |  |  |  |  |  |  |  |
| X4 | 23 | 40.22 ± 1.7 | 41.11 ± 2 | 22.45 ± 3.4 | 84 ± 16 | 14 ± 1 | 6.6 ± 2.5 | 2 | 82 | 81 | 0.52 |
| X5 | 205 | 36.88 ± 1.1 | 38.97 ± 2.2 | 17.48 ± 2.5 | 101 ± 1. | 14 ± 2 | 7.69 ± 2.5 | 2.8 ± 0.4 | 89 | 89 | 0.54 |
| P54 | 20 | 31.57 ± 1.2 | 38.77 ± 1.3 | 16.53 ± 1.5 | 121 ± 0.5 | 18 ± 2 | 17.75 ± 3.5 | 2.4 ± 0.5 | 71 | 70 | 0.39 |
|  |  |  |  |  |  |  |  |  |  |  |  |
| ***Ros*** |  |  |  |  |  |  |  |  |  |  |  |
| X6 | 20 | 35.75 ± 2 | 36.07 ± 2.7 | 18.43 ± 2.7 | 103 ± 1.3 | 44 ± 6 | 84.14 ± 14.6 | 3.6 ± 0.5 | 94 | 94 | 0.68 |
| P56 | 195 | 35.25 ± 2.2 | 38.78 ± 4.1 | 21.53 ± 3.7 | 123 ± 1.1 | 46 ± 3 | 96.62 ± 11.4 | 3.2 ± 0.8 | 82 | 82 | 0.60 |
| P57 | 8.5 | 35.5 ± 1.2 | 39.82 ± 3.6 | 21.64 ± 4 | 152 ± 3.2 | 47 ± 8 | 132.09 ± 40 | 3 ± 0.7 | 82 | 82 | 0.62 |
|  |  |  |  |  |  |  |  |  |  |  |  |
| ***Ros Gruixat*** |  |  |  |  |  |  |  |  |  |  |  |
| X7 | 20 | NA | NA | NA | NA | NA | NA | NA | 68 | 67 | 0.71 |
| P58 | 285 | 35.82 ± 2.1 | 37.59 ± 4.7 | 21.84 ± 4.9 | 148 ± 21 | 43 ±8 | 119.96 ± 28.7 | 2.6 ± 0.5 | 76 | 76 | 0.56 |
| P59 | NA | NA | NA | NA | NA | NA | NA | NA | 62 | 59 | 0.49 |
| P60 | 19 | 37.17 ± 3.7 | 39.63 ± 3.5 | 23.58 ± 5.4 | 140 ± 16 | 45 | 109.35 ± 22.8 | 2.4 ± 0.5 | 69 | 68 | 0.76 |
|  |  |  |  |  |  |  |  |  |  |  |  |
| ***Ros Prim*** |  |  |  |  |  |  |  |  |  |  |  |
| P61 | 215 | 37.61 ± 2.3 | 39.12 ± 3.1 | 22.38 ± 3.6 | 142 ± 12 | 44 ± 3 | 112.4 ± 8.9 | 2.8 ± 0.4 | 58 | 56 | 0.62 |
| P62 | 22 | 33.88 ± 1.3 | 39.75 ± 2.2 | 22.7 ± 3.6 | 143 ± 29 | 48 ± 2 | 123.73 ± 30.4 | 3 | 43 | 43 | 0.69 |
|  |  |  |  |  |  |  |  |  |  |  |  |
| ***Tap de Cortí*** |  |  |  |  |  |  |  |  |  |  |  |
| P63 | 17 | NA | NA | NA | NA | NA | NA | NA | 83 | 82 | NA |
| P64 | 16 | 32.66 ± 2.7 | 32.54 ± 2.3 | 14.15 ± 2.4 | 54 ± 5 | 2.68 ± 1 | 17.17 ± 1.5 | 2.8 ± 0.4 | NA | NA | 0.55 |
| P65 | 205 | 32.93 ± 1.1 | 32.1 ± 2.4 | 12.88 ± 2.2 | 65 ± 5 | 2.86 ± 2 | 24.66 ± 1.7 | 2.4 ± 0.5 | 75 | 75 | 0.67 |
| P66 | 18 | 33.78 ± 2 | 31.95 ± 2.6 | 14.12 ± 1.2 | 61 ± 2 | 2.9 ± 1 | 23.48 ± 2.2 | 2.6 ± 0.5 | NA | NA | 0.49 |
| P67 | 205 | 33.84 ± 0.8 | 33.34 ± 1.8 | 14.83 ± 1.4 | 65 ± 3 | 2.84 ± 3 | 21.93 ± 4 | 2.4 ± 0.5 | 86 | 86 | 0.51 |
|  |  |  |  |  |  |  |  |  |  |  |  |
| ***Reference accessions*** |  |  |  |  |  |  |  |  |  |  |  |
| California Wonder | 245 | 59.12 ± 3.3 | -0.08 ± 2.5 | 56.24 ± 3 | 72 ± 4 | 62 ± 4 | 93.15 ± 8.1 | 3 | 07 | 05 | 0.78 |
| Chile Serrano | 85 | 41.93 ± 2.4 | 40.5 ± 1.1 | 26.95 ± 4 | 36 ± 4 | 13 ± 1 | 3.24 ± 1 | 2.4 ± 0.5 | 79 | 78 | 0.60 |
| Bola | 435 | 36.5 ± 1.3 | 35.59 ± 2.7 | 18.86 ± 1.5 | 33 ± 2 | 27 ± 3 | 14.61 ± 5.4 | 2.6 ± 0.5 | 39 | 26 | 0.70 |
| Pasilla Bajío | 485 | 28.03 ± 0.8 | 3.12 ± 1.1 | 3.73 ± 0.8 | 155 ± 12 | 29 ± 2 | 27.06 ± 7.7 | 2.2 ± 0.4 | 68 | 66 | 1.29 |
| Serrano Criollo de Morelos | 23 | 39.9 ± 2.6 | 39.83 ± 1.7 | 23.65 ± 2.4 | 101 ± 9 | 2 ± 1 | 16.52 ± 4.3 | 2.2 ± 0.4 | 88 | 83 | 0.74 |
| Piquillo | 25 | 36.13 ± 1.9 | 34.63 ± 3.5 | 17.2 ± 2.9 | 71 ± 9 | 41 ± 3 | 41.8 ± 9.3 | 2.6 ± 0.9 | 81 | 76 | 0.77 |

^1^ SL: stem length (mm), FL: fruit colour lightness, FA: fruit colour green/red, FB: fruit colour blue/yellow, FLE: fruit length (mm), FWI: fruit width (mm), FW: fruit weight (g), NL: number of locules, PMV: pollen morphological viability (%), PBV: pollen biological viability (%), SW: seed weight (g).
