# Supplementary material for: Genetic diversity, population structure, and phylogeny of insular Spanish pepper landraces (Capsicum annuum L.) through phenotyping and genotyping-by-sequencing
Source: Front Plant Sci. 2024 Oct 30;15:1435427. doi: 10.3389/fpls.2024.1435427 (PMC11557316; doi:10.3389/fpls.2024.1435427)
Supplement: Supplementary file 3 [file Table3.pdf]

Supplementary Table 3. Observed (O) and expected (E) number of heterozygous sites, fixation index (F), and estimated heterozygosity values (Het, %) for all the studied accessions.

Accession	O	E	F	Het (%)
Pebrera Blanca				
PB_P1	6,588	72,475	0.91	0.55
PB_P2	9,127	72,463	0.87	0.76
PB_P3	8,898	72,479	0.88	0.74
PB_P4	9,067	72,465	0.87	0.76
PB_P5	7,470	72,482	0.90	0.62
PB_P6	11,611	72,473	0.84	0.97
PB_P7	8,942	72,463	0.88	0.75
PB_P8	8,603	72,477	0.88	0.72
PB_P9	8,708	72,477	0.88	0.73
PB_P10	8,128	72,470	0.89	0.68
PB_P11	9,620	72,473	0.87	0.80
PB_P12	7,696	72,472	0.89	0.64
PB_P13	10,927	72,486	0.85	0.91
PB_P14	7,096	72,485	0.90	0.59
PB_P15	8,139	72,472	0.89	0.68
PB_P16	7,507	72,481	0.90	0.63
PB_P17	6,605	72,497	0.91	0.55
PB_P18	11,201	72,448	0.85	0.94
PB_P19	12,753	72,461	0.82	1.07
PB_P20	11,766	72,468	0.84	0.98
PB_P21	9,144	72,476	0.87	0.76
PB_P22	11,697	72,469	0.84	0.98
PB_P23	13,603	72,453	0.81	1.14
PB_P24	11,225	72,471	0.85	0.94
PB_P25	9,775	72,470	0.87	0.82
PB_P26	12,747	72,459	0.82	1.06
Citró de Matances				
CdM_P27	11,445	72,465	0.84	0.96
CdM_P28	10,722	72,466	0.85	0.90
CdM_P29	10,067	72,470	0.86	0.84
CdM_P30	13,320	72,462	0.82	1.11
CdM_P31	9,012	72,479	0.88	0.75
CdM_P32	7,499	72,494	0.90	0.63
CdM_P33	6,807	72,486	0.91	0.57
CdM_P34	6,750	72,484	0.91	0.56
CdM_P35	8,971	72,491	0.88	0.75
CdM_P36	10,831	72,483	0.85	0.90
CdM_P37	6,108	72,496	0.92	0.51

Accession	O	E	F	Het (%)
Citró de Matances				
CdM_P38	7,649	72,498	0.89	0.64
CdM_P39	5,786	72,492	0.92	0.48
CdM_P41	4,917	72,500	0.93	0.41
CdM_P42	6,772	72,486	0.91	0.57
CdM_P43	4,584	72,503	0.94	0.38
CdM_P44	5,272	72,497	0.93	0.44
CdM_P45	5,779	72,492	0.92	0.48
CdM_P46	8,373	72,497	0.88	0.70
CdM_P47	4,798	72,499	0.93	0.40
Banya de Cabra				
BdC_P48	9,032	72,478	0.88	0.72
BdC_P49	10,097	72,478	0.86	1.03
Blau				
B_P50	9,247	72,472	0.87	0.84
B_P51	8,985	72,478	0.88	0.77
Cirereta				
C_P52	7,588	72,488	0.90	0.82
D'Envinagrar				
DE_P53	9,865	72,482	0.86	1.95
Fulla d'Olivera				
FO_P54	6,699	72,491	0.91	0.69
FO_P55	8,594	72,479	0.88	0.86
Ros				
R_P56	8,640	72,467	0.88	0.75
R_P57	10,812	72,468	0.85	0.74
Ros Gruixat				
RG_P58	12,384	72,474	0.83	0.75
RG_P59	9,157	72,483	0.87	0.77
RG_P60	9,166	72,471	0.87	0.90
Ros Prim				
RP_P61	9,179	72,471	0.87	0.72
RP_P62	8,824	72,472	0.88	0.76
Tap de Cortí				
TdC_P63	8,215	72,485	0.89	0.63
TdC_P64	10,288	72,474	0.86	0.56
TdC_P65	23,380	72,469	0.68	0.77
TdC_P66	11,042	72,479	0.85	0.92
TdC_P67	7,768	72,487	0.89	0.65
